# Analysis of Inactivation of SARS-CoV-2 by Specimen Transport Media, Nucleic Acid Extraction Reagents, Detergents, and Fixatives

**DOI:** 10.1128/JCM.01713-20

**Published:** 2020-10-21

**Authors:** Stephen R. Welch, Katherine A. Davies, Hubert Buczkowski, Nipunadi Hettiarachchi, Nicole Green, Ulrike Arnold, Matthew Jones, Matthew J. Hannah, Reah Evans, Christopher Burton, Jane E. Burton, Malcolm Guiver, Patricia A. Cane, Neil Woodford, Christine B. Bruce, Allen D. G. Roberts, Marian J. Killip

**Affiliations:** a High Containment Microbiology, NIS Laboratories, National Infection Service, Public Health England, Colindale, London, United Kingdom; b UK Public Health Rapid Support Team, Public Health England/London School of Hygiene and Tropical Medicine, London, United Kingdom; c Virology Department, Clinical Sciences Laboratory, National Infection Service, Public Health England, Manchester Public Health Laboratory, Manchester, United Kingdom; d NIS Laboratories, National Infection Service, Public Health England, Colindale, London, United Kingdom; Cepheid

**Keywords:** COVID-19, SARS-CoV-2, coronavirus, inactivation, safety testing, specimen transport tubes, molecular extraction reagents, lysis buffers, clinical diagnostics, biosafety, diagnostics

## Abstract

The COVID-19 pandemic has necessitated a multifaceted rapid response by the scientific community, bringing researchers, health officials, and industry together to address the ongoing public health emergency. To meet this challenge, participants need an informed approach for working safely with the etiological agent, the novel human coronavirus SARS-CoV-2. Work with infectious SARS-CoV-2 is currently restricted to high-containment laboratories, but material can be handled at a lower containment level after inactivation. Given the wide array of inactivation reagents that are being used in laboratories during this pandemic, it is vital that their effectiveness is thoroughly investigated. Here, we evaluated a total of 23 commercial reagents designed for clinical sample transportation, nucleic acid extraction, and virus inactivation for their ability to inactivate SARS-CoV-2, as well as seven other common chemicals, including detergents and fixatives. As part of this study, we have also tested five filtration matrices for their effectiveness at removing the cytotoxic elements of each reagent, permitting accurate determination of levels of infectious virus remaining following treatment. In addition to providing critical data informing inactivation methods and risk assessments for diagnostic and research laboratories working with SARS-CoV-2, these data provide a framework for other laboratories to validate their inactivation processes and to guide similar studies for other pathogens.

## Abstract (precis)

The COVID-19 pandemic has necessitated a multifaceted rapid response by the scientific community, bringing researchers, health officials, and industry together to address the ongoing public health emergency. To meet this challenge, participants need an informed approach for working safely with the etiological agent, the novel human coronavirus SARS-CoV-2. Work with infectious SARS-CoV-2 is currently restricted to high-containment laboratories, but material can be handled at a lower containment level after inactivation.

## INTRODUCTION

Infection with the novel human betacoronavirus SARS-CoV-2 can cause a severe or fatal respiratory disease, termed COVID-19 (1–3). As the COVID-19 pandemic has developed, millions of clinical samples have been collected for diagnostic evaluation. SARS-CoV-2 has been classified as a hazard group 3 pathogen, and, as such, any work with infectious virus must be carried out in high-containment laboratories (containment level 3 [CL3] in the United Kingdom) with associated facility, equipment, and staffing restrictions. Guidance from Public Health England (PHE), the World Health Organization (WHO), and the U.S. Centers for Disease Control and Prevention (CDC) enables nonpropagative testing of clinical specimens to be carried out at the lower CL2 or biosafety level 2 (BSL-2), with the requirements that noninactivated material is handled within a microbiological safety cabinet (MSC) and that the process has been suitably and sufficiently risk assessed (4–6). An exception to this is for point-of-care (POC) or near-POC testing, which WHO and CDC biosafety guidelines allow to be performed outside an MSC when a local risk assessment so dictates and appropriate precautionary measures are in place (5, 6). To allow safe movement of clinical samples from CL3/BSL-3 laboratories to CL2/BSL-2, virus inactivation procedures need to be validated, and formal validation of these protocols is often an operational requirement for clinical and research laboratories handling SARS-CoV-2.

Efficacy of virus inactivation depends on numerous factors, including the nature and concentration of pathogen, sample matrix, concentration of inactivation agent(s), and contact time. To date, there are limited data on the efficacy of SARS-CoV-2-specific inactivation approaches in the scientific literature, and risk assessments have largely been based upon inactivation information for genetically related coronaviruses. Previous studies have found that treatment with heat, chemical inactivants, UV light, gamma irradiation, and a variety of detergents is effective at inactivating the high-consequence human coronaviruses SARS-CoV-1 and Middle East respiratory syndrome coronavirus (MERS-CoV) (7–13). However, limited validation data exist for coronavirus inactivation by sample transport reagents used to store clinical samples after collection and commercial molecular extraction lysis buffers used in steps prior to nucleic acid extraction for diagnostic testing. Furthermore, the precise composition of many commercial reagents is proprietary, preventing ingredient-based inference of inactivation efficacies between reagents. Some limited preliminary data on SARS-CoV-2 inactivation by heat (14, 15) or chemical (16–21) treatments are available, but given the current level of diagnostic and research activities, there is an urgent need to comprehensively investigate the SARS-CoV-2-specific inactivation efficacy of available methods to support safe virus handling.

An important consideration in inactivation efficacy assay development is cytotoxicity, a typical effect of many chemical inactivants. To mitigate cytotoxic effects, the inactivation agent needs to be either diluted out or removed from treated samples prior to testing for infectious virus. Each of these methods for addressing cytotoxicity presents its own challenges. Sample dilution requires the use of high-titer stocks of virus (e.g., >10^8^ PFU/ml) to be able to demonstrate a significant titer reduction and reduces recovery of low-level residual virus from treated samples, making it difficult or impossible to distinguish complete from incomplete virus inactivation. In contrast, methods for purification of virus away from cytotoxic components in treated samples may also remove virus or affect virus viability. Accurate quantification of remaining infectious virus ideally requires complete removal of cytotoxicity without compromising assay sensitivity, which needs careful consideration of reagent and purification processes prior to the performance of inactivation tests.

Here, we describe optimal methods for the removal of cytotoxicity from samples treated with commercial reagents, detergents, and fixatives. These data were then used in evaluations of the effectiveness of these chemicals for inactivating SARS-CoV-2. This work, applicable to both diagnostic and research laboratories, provides invaluable information for public health and basic research responses to the COVID-19 pandemic by supporting safe approaches for collection, transport, extraction, and analysis of SARS-CoV-2 samples. Furthermore, our studies investigating purification of a wide range of cytotoxic chemicals are highly applicable to inactivation studies for other viruses, thereby supporting rapid generation of inactivation data for known and novel viral pathogens.

## MATERIALS AND METHODS

### Cells and virus.

Vero E6 cells (Vero C1008; ATCC CRL-1586) were cultured in modified Eagle's minimum essential medium (MEM) supplemented with 10% (vol/vol) fetal calf serum (FCS). Virus used was the SARS-CoV-2 strain hCOV-19/England/2/2020, isolated by PHE from the first patient cluster in the United Kingdom on 29 January 2020. This virus was obtained at passage 1 and used for inactivation studies at passage 2 or 3. All infectious work was carried out using a class III microbiology safety cabinet (MSCIII) in a CL3 laboratory. Working virus stocks were generated by infecting Vero E6 cells at a multiplicity of infection (MOI) of 0.001 in the presence of 5% FCS. Cell culture supernatants were collected at 72 h postinfection, clarified for 10 min at 3,000 × *g*, aliquoted, and stored at −80°C until required. Viral titers were calculated by either plaque assays or 50% tissue culture infectious doses (TCID_50_). For plaque assays, 24-well plates were seeded the day before the assay (1.5 × 10^5^ cells/well in MEM–10% FCS). Tenfold dilutions of virus stock were inoculated onto plates (100 μl per well) at room temperature for 1 h, overlaid with 1.5% medium-viscosity carboxymethylcellulose (Sigma-Aldrich), and incubated at 37°C in 5% CO_2_ for 3 days. For TCID_50_, 10-fold dilutions of virus stock (25 μl) were plated onto 96-well plates containing Vero E6 cell suspensions (2.5 × 10^4^ cells/well in 100 μl of MEM–5% FCS) and incubated at 37°C in 5% CO_2_ for 5 to 7 days. Plates were fixed with 4% (vol/vol) formaldehyde–phosphate-buffered saline (PBS) and stained with 0.2% (vol/vol) crystal violet-water. TCID_50_ titers were determined by the Spearman-Kärber method (22, 23).

### Reagents and chemicals used for SARS-CoV-2 inactivation.

The commercial reagents evaluated in this study, along with their compositions (if known) and manufacturers’ instructions for use (if provided), are given in Table S1 in the supplemental material. Specimen transport reagents tested were the following: Sigma molecular transport medium (MM; Medical Wire), eNAT (Copan), Primestore molecular transport medium (MTM; Longhorn Vaccines and Diagnostics), Cobas PCR medium (Roche), Aptima specimen transport medium (Hologic), DNA/RNA Shield (Zymo Research), guanidine hydrochloride (GCHl) and guanidine thiocyanate (GITC) buffers containing Triton X-100 (both, Oxoid/Thermo Fisher), and virus transport and preservation medium (inactivated) (BioComma). Molecular extraction reagents tested were the following: AVL, RLT, and AL (all Qiagen); MagNA Pure external lysis buffer and Cobas Omni LYS used for on-board lysis by Cobas extraction platforms (Roche); viral PCR sample solution (VPSS) and lysis buffer (both, E&O Laboratories); NeuMoDx lysis buffer (NeuMoDx Molecular); Samba II SCoV lysis buffer (Diagnostics for the Real World); NucliSENS lysis buffer (bioMérieux); Panther Fusion specimen lysis tubes (Hologic); and an in-house extraction buffer containing guanidine thiocyanate and Triton X-100 (PHE Media Services). Detergents tested were the following: Tween 20, Triton X-100, and NP-40 Surfact-Amps detergent solution (all, Thermo Scientific) and UltraPure SDS 10% solution (Invitrogen). Other reagents assessed included polyhexamethylene biguanide (PHMB; Blueberry Therapeutics), formaldehyde and glutaraldehyde (both, TAAB), and ethanol and methanol (both, Fisher Scientific).

### Removal of reagent cytotoxicity.

Specimen transport tube reagents were assessed undiluted unless otherwise indicated. For testing of molecular extraction reagents, mock samples were generated by diluting reagent in PBS at ratios given in the manufacturers’ instructions. Detergents, fixatives, and solvents were all assessed at the concentrations indicated in Table 1. All methods were evaluated in a spin column format for ease of sample processing within the high-containment laboratory. Pierce detergent removal spin columns (0.5 ml; Thermo Scientific), Microspin Sephacryl S400HR (GE Healthcare), and Amicon Ultra 0.5-ml 50-kDa centrifugal filters (Merck Millipore) were prepared according to the manufacturers’ instructions. Sephadex LH-20 (GE Healthcare) and Bio-Beads SM2 resin (Bio-Rad) were suspended in PBS and poured into empty 0.8-ml Pierce centrifuge columns (Thermo Scientific) and centrifuged for 1 min at 1,000 × *g* to remove PBS immediately before use. For all matrices aside from the Amicon Ultra columns, 100 μl of treated sample was added to each spin column, incubated for 2 min at room temperature, and then eluted by centrifugation at 1,000 × *g* for 2 min. For Amicon Ultra filters, 500 μl of sample was added and centrifuged at 14,000 × *g* for 10 min, which was followed by three washes with 500 μl of PBS. Sample was then collected by resuspending contents of the filtration device with 500 μl of PBS. To assess remaining cytotoxicity, a 2-fold dilution series of treated filtered sample was prepared in PBS, and 6.5 μl of each dilution was transferred in triplicate to 384-well plates containing Vero E6 cells (6.25 × 10^3^ cells/well in 25 μl of MEM–5% FCS) and incubated overnight. Cell viability was determined by a CellTiter Aqueous One Solution cell proliferation assay (Promega) according to the manufacturer’s instructions. Normalized values of absorbance (relative to untreated cells) were used to fit a four-parameter equation to semilog plots of the concentration-response data and to interpolate the concentration that resulted in 80% cell viability (20% cytotoxic concentration [CC_20_]) in reagent-treated cells. All analyses were performed using GraphPad Prism (version 8.4.1; GraphPad Software).

**TABLE 1:** Purification of reagents

Reagent type and name^*a*^	Reagent/medium ratio or % (vol/vol) tested	Postfiltration dilution factor of eluate needed for CC_20_ by method (95% CI)^*b*^					
		Unpurified	Sephadex LH-20	Sephacryl S400HR	Amicon Ultra 50-kDa	Pierce DRSC	Bio-Beads SM2
Specimen transport tube reagents							
BioComma	Undiluted	36.2 (30.1–44.0)	<2 (NA)	<2 (NA)	<1 (NA)	<1 (NA)	12.1 (9.2–16.4)
Sigma MM	1.5:1	417 (306–619)	59.2 (51.8–67.1)	48.7 (44.6–53.3)	4.0 (3.6–4.3)	<1 (NA)	7.6 (6.5–8.9)
eNAT	3:1	70.1 (55.0–88.5)	<1 (NA)	2.8 (2.5–3.1)	<1 (NA)	<1 (NA)	24.4 (20.2–30.2)
Primestore MTM	3:1	56.2 (47.2–66.3)	<1 (NA)	4.8 (NC)	<1 (NA)	<1 (NA)	18.3 (15.4–22.1)
Cobas PCR medium	1:1	55.5 (46.5–67.5)	2.7 (2.3–3.1)	5.2 (4.6–5.9)	<1 (NA)	<1 (NA)	26.5 (23.5–30.2)
Aptima STM	Undiluted	178 (<178–204)	<1 (NA)	32.0 (NC)	7.6 (NC)	<1 (NA)	<1 (NA)
DNA/RNA Shield	Undiluted	1,098 (994–1,231)	1,155 (1,076–1,253)	82.3 (<82.3–94.7)	29.6 (26.2–32.3)	66.1 (58.1–75.8)	7.1 (5.5–8.6)
40% GHCl/Tx TM	Undiluted	245 (205–288)	24.5 (<24.5–31.5)	25.9 (<25.9–36.7)	13.3 (<13.3–15.6)	2.2 (NC)	119 (103–135)
2M GITC/Tx TM	Undiluted	245 (215–277)	19.4 (<19.4–23.9)	19.1 (15.4–26.3)	37.8 (NC)	<1 (NA)	127 (113–141)
4 M GITC/Tx TM	Undiluted	1,054 (889–1,262)	545 (487–613)	141 (102–201)	211 (172–247)	3.5 (3.1–3.9)	20.3 (15.2–27.9)
Molecular extraction reagents							
Buffer AVL	4:1	61.6 (50.8–75.1)	<1 (NA)	3.2 (2.9–3.5)	<1 (NA)	<1 (NA)	26.1 (21.5–32.3)
MagNA Pure LB	1:1	1,934 (1,348–2,780)	1,391 (<1,391–1,654)	474 (434–517)	346 (<346–382)	59.1 (45.6–70.4)	5.8 (1.4–7.8)
NucliSENS	1:1	60.5 (54.9–66.2)	<1 (NA)	4.3 (4.0–4.9)	<1 (NA)	<1 (NA)	4.6 (<4.6–6.7)
Panther Fusion	1.42:1	196 (<196–214)	<1 (NA)	18.0 (<18.0–19.4)	15.9 (<15.9–16.5)	<1 (NA)	<1 (NA)
Buffer AL	1:1	61.9 (56.7–65.4)	37.4 (34.7–41.1)	7.8 (6.6–9.3)	30.5 (25.5–36.3)	29.5 (25.9–33.9)	16.5 (14.6–18.9)
Cobas Omni LYS	1:1	225 (<225–255)	142 (NC)	45.8 (<45.8–55.6)	117 (NC)	16.7 (NC)	6.7 (2.9–8.7)
PHE in-house LB	4:1	231 (<231–310)	26.2 (22.0–31.8)	11.4 (9.9–13.2)	2.7 (<2.7–4.9)	<1 (NA)	12.9 (9.8–17.9)
NeuMoDx LB	1:1	30.2 (24.1–37.9)	8.0 (7.3–8.8)	2.0 (1.7–2.4)	7.5 (6.6–8.1)	4.2 (0.4–6.9)	6.8 (<6.8–8.4)
E&O Labs VPSS	Undiluted	174 (145–206)	24.9 (22.1–28.4)	14.2 (11.7–17.5)	7.7 (<7.7–14.5)	<1 (NA)	11.7 (8.5–16.4)
E&O Lab LB	Undiluted	69.0 (62.7–76.9)	9.5 (<9.5–11.0)	8.0 (7.4–8.7)	2.2 (NC)	<1 (NA)	4.1 (3.5–4.7)
Samba II SCoV LB	Undiluted	177 (<177–213)	68.2 (63.0–75.4)	27.3(24.2–30.1)	5.2 (<5.2–6.0)	<1 (NA)	1.5 (1.0–1.8)
Buffer RLT	Undiluted	48.0 (40.3–58.0)	2.9 (2.3–4.3)	<1 (NA)	<1 (NA)	<1 (NA)	18.5 (15.3–22.8)
Detergents							
Triton X-100	1%	185 (<185–211)	48.4 (<48.4–58.4)	∼17.22 (NC)	<1 (NA)	<1 (NA)	<1 (NA)
Tween 20	1%	7.7 (6.9–8.6)	4.2 (<3.8–4.9)	1.3 (1.0–1.7)	4.4 (4.0–5.1)	4.9 (3.4–7.5)	<1 (NA)
SDS	1%	69.6 (NA)	<1 (NA)	<1 (NA)	<1 (NA)	<1 (NA)	<1 (NA)
NP-40	1%	320 (<320–402)	171 (<171–196)	140 (123–161)	<1 (NA)	<1 (NA)	<1 (NA)
Other							
Formaldehyde	4%	4207 (3,270–5,844)	288 (226–383)	111 (93–136)	<1 (NA)	51.6 (<51.6–65.9)	1,309 (1,058–1,685)
Formaldehyde + glutaraldehyde	2% + 1.5%	4227 (3,183–6,027)	39.8 (32.7–51.4)	97.9 (82.9–118)	<1 (NA)	22.6 (<22.6–27.2)	1,545 (1,164–2,203)
Ethanol	100%	63.3 (27.6–103)	<1 (NA)	<1 (NA)	<1 (NA)	<1 (NA)	8.8 (6.5–12.5)
Methanol	100%	108 (79.5–155)	<1 (NA)	<1 (NA)	<1 (NA)	<1 (NA)	2.2 (1.9–2.5)
0.1% PHMB	0.1%	30.1 (26.6–34.2)	9.5 (8.9–10.2)	<1 (NA)	<1 (NA)	<1 (NA)	9.8 (<9.8–11.8)
1.0% PHMB	1%	328 (304–356)	132 (111–154)	<1 (NA)	<1 (NA)	9.3 (<9.3–11.1)	203 (<203–299)
2.0% PHMB	2%	837 (<837–1,141)	240 (198–282)	4.1 (3.7–4.5)	<1 (NA)	25.0 (<20.9–29.0)	479 (<479–647)

a LB, lysis buffer; STM, specimen transport medium; TM, transport medium; MM, molecular transport medium; GHCl, guanidine hydrochloride; GITC, guanidinium isothiocyanate; Tx, Triton X-100; PHMB, polyhexamethylene biguanide.

b Values represent the dilution factor required after one purification process to achieve the CC_20_. CI, confidence interval; NA, not available; NC, not able to be calculated.

### SARS-CoV-2 inactivation.

For commercial products, virus preparations (tissue culture fluid, titers ranging from 1 × 10^6^ to 1 × 10^8^ PFU/ml) were treated in triplicate with reagents at concentrations and for contact times recommended in the manufacturers’ instructions for use, where available, or for concentrations and times specifically requested by testing laboratories. Where a range of concentrations was given by the manufacturer, the lowest ratio of product to sample was tested (i.e., lowest recommended concentration of test product). Specimen transport tube reagents were tested using a ratio of 1 volume of tissue culture fluid to 10 volumes of reagent, unless a volume ratio of sample fluid to reagent was specified by the manufacturer. Detergents, fixatives, and solvents were tested at the concentrations and times indicated in the tables. All inactivation steps were performed at ambient room temperature (18 to 25°C). For testing of alternative sample types, virus was spiked into the indicated sample matrix at a ratio of 1:9 and then treated with test reagents as described above. All experiments included triplicate control mock-treated samples with equivalent volumes of PBS in place of test reagent. Immediately following the required contact time, 1 ml of treated sample was processed using the appropriately selected filtration matrix. Reagent removal for inactivation testing was carried out in a larger spin column format using Pierce 4-ml detergent removal spin columns (Thermo Fisher) or by filling empty Pierce 10-ml-capacity centrifuge columns (Thermo Fisher) with SM2 Bio-Beads, Sephacryl S400HR, or Sephadex LH-20 to give 4 ml of packed beads/resin. For purification using Amicon filters, two 500-μl samples were purified using two centrifugal filters by the method previously described and then pooled. For formaldehyde and formaldehyde with glutaraldehyde removal, one filter was used with one 500-μl sample volume, which was resuspended after processing in 500 μl of PBS and added to 400 μl of MEM–5% FBS. For inactivation of infected monolayers, 12.5-cm^2^ flasks of Vero E6 cells (2.5 × 10^6^ cells/flask in 2.5 ml of MEM–5% FBS) were infected at an MOI of 0.001 and incubated at 37°C in 5% CO_2_ for 24 h. Supernatant was removed, and cells were fixed using 5 ml of formaldehyde or formaldehyde and glutaraldehyde at room temperature for 15 or 60 min. The fixative was removed, and monolayers were washed three times with PBS before cells were scraped into 1 ml of MEM–5% FBS and sonicated (three cycles of 10 s on and 10 s off at 100% power and amplitude) using a UP200St with VialTweeter attachment (Hielscher Ultrasound Technology). Supernatants were clarified by centrifugation at 3,000 × *g* for 10 min.

### SARS-CoV-2 quantification and titer reduction evaluation.

Virus present in treated and purified samples or in mock-treated and purified samples was quantified by either TCID_50_ or plaque assay. As additional assay controls, unfiltered mock-treated sample was titrated to determine virus loss during filtration, and filtered test reagent-only (no virus) sample was titrated to determine residual test buffer cytotoxicity. For TCID_50_ assays, neat to 10^−7^ 10-fold dilutions were prepared, and for plaque assays, neat to 10^−5^ 10-fold dilutions were prepared, both in MEM–5% FCS. TCID_50_ titers were determined by the Spearman-Kärber method (22, 23). When the levels of detected virus were so low that the TCID_50_ could not be calculated by the Spearman-Kärber method, the TCID_50_ was calculated by the Taylor method (24). When no virus was detectable, values are given as less than or equal to the Taylor-derived TCID_50_ titer given by a single virus-positive well at the lowest dilution at which no cytotoxicity was observed. Titer reduction was calculated by subtracting the mean logarithmic virus titer for test buffer-treated, purified conditions from the mean logarithmic virus titer for the PBS-treated, purified condition, with standard errors calculated according to Spearman (22).

### Serial passages of treated samples.

In parallel to virus quantification, 12.5-cm^2^ flasks of Vero E6 cells (6.25 × 10^4^ cells/flask in 2.5 ml of MEM–5% FBS) were inoculated with either 500 μl or 50 μl of treated filtered sample. Flasks were examined for cytopathic effect (CPE), and 500 μl of culture medium from each flask was used to inoculate new 12.5-cm^2^ flasks of Vero E6 cells after 7 days. If no CPE was observed, this process was continued for up to four serial passages. For the duration of the passage series, a flask of untreated cells was included as a control for cross-contamination between flasks, and a SARS-CoV-2-infected control was included to ensure suitable conditions for virus propagation. To distinguish CPE from any residual cytotoxicity associated with test reagents, samples of cell culture medium were taken from each flask at the beginning and end of each passage. Nucleic acid was extracted from cell culture medium manually using a QIAamp Viral RNA Minikit (Qiagen) or by using NucliSENS easyMAG or EMAG platforms (both, bioMérieux). Viral RNA levels were quantified by quantitative reverse transcriptase PCR (qRT-PCR) specific for the SARS-CoV-2 E gene (25) using TaqMan Fast 1-Step Master Mix (Applied Biosystems) on a 7500 Fast real-time PCR system (Applied Biosystems). A positive result for virus amplification was recorded if effects on the monolayer consistent with CPE and a decrease in the threshold cycle (*C_T_*) value across the course of a passage were observed.

## RESULTS

### Reagent filtration optimization to minimize cytotoxicity and maximum virus recovery.

**FIG 1:**
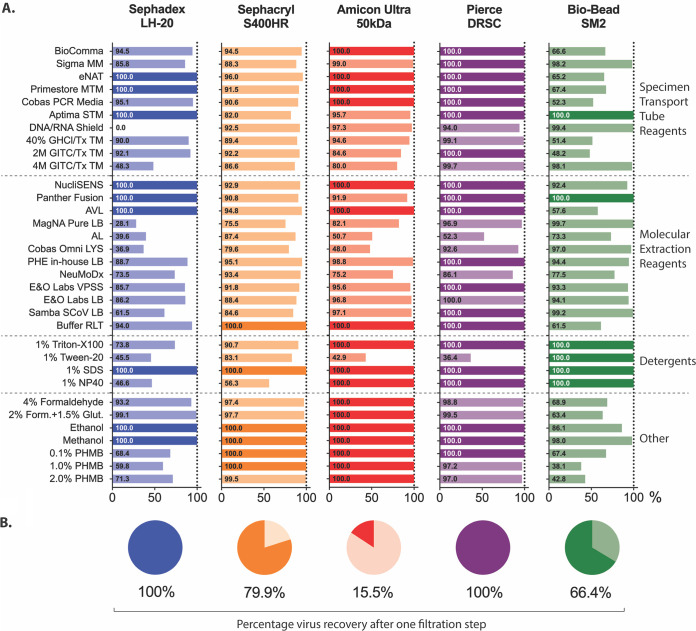
Effectiveness of five filtration matrices at removing cytotoxicity. (A) SARS-CoV-2 virus in clarified cell culture supernatant was treated with indicated reagent for 2 min at room temperature before being purified through one of five filtration matrices: Sephadex LH-20, Sephacryl S400HR, Amicon Ultra 50-kDa-molecular-weight cutoff, Pierce detergent removal spin columns (DRSC), or Bio-Bead SM2. Values indicate the percent toxicity removal after one purification cycle relative to levels in unpurified samples (based on CC_20_ values). More details are provided in Table 1. (B) Percentage of input virus remaining in eluate after one purification cycle through each filtration matrix. GHCl, guanidine hydrochloride; GITC, guanidinium isothiocyanate; Tx, Triton X-100; PHMB, polyhexamethylene biguanide; SDS, sodium dodecyl sulfate; NP-40, nonyl phenoxypolyethoxylethanol; LB, lysis buffer; TM, transport medium.

Prior to evaluating the effectiveness of the reagents at inactivating SARS-CoV-2, we investigated the cytotoxicity of each reagent before and after filtration though one of five matrices: Sephadex LH-20, Sephacryl S400HR, Amicon Ultra 50- kDa-molecular-weight-cutoff centrifugal filters, Pierce detergent removal spin columns (DRSC), and Bio-Beads SM2 nonpolar polystyrene adsorbents. Reagents were diluted with PBS to the working concentrations recommended by the manufacturer (for commercial sample transport and molecular extraction reagents) or to the concentrations indicated in the tables (for all other chemicals), followed by a single reagent removal step with each filtration matrix. Dilution series of filtered and unfiltered samples were generated to determine concentration-dependent cytotoxicity, from which the CC_20_ value for each combination of reagent and filtration method was interpolated (see Fig. S1 in the supplemental material). The CC_20_ was chosen as cells retain 80% viability at this concentration and enable distinction of active SARS-CoV-2 replication by visualization of CPE in the monolayer. Table 1 shows the dilution factor of reagent-treated sample required to achieve the CC_20_ after filtration, with a value of <1 indicating complete removal of cytotoxicity. These data were used to determine the relative cytotoxicity removed by one filtration step for each combination of reagent and matrix (Fig. 1A).

All unfiltered reagents tested here were cytotoxic, but the degree of cytotoxicity varied considerably as did the optimal filtration matrix for each reagent. The detergent Tween 20 used at a 1% concentration was the least cytotoxic unfiltered, requiring a dilution factor of only 7.7 to reach the CC_20_ although only the Bio-Bead SM2 filters were effective at removing all cytotoxicity. The chemical fixative combination of 2% formaldehyde and 1.5% glutaraldehyde was the most cytotoxic unfiltered, requiring a dilution of over 4,000 to reach the CC_20_, with only the Amicon Ultra columns able to remove 100% of the cytotoxicity. However, for the majority reagents (27/34) tested, filtration through at least one matrix type removed 100% of cytotoxicity, allowing neat eluate to be used directly in cell culture without further dilution. There were several exceptions to this: DNA/RNA Shield (maximum 99.4% cytotoxicity removal using SM2), 40% GHCl (99.1% using Pierce DRSC), 4 M GITC (99.7% using Pierce DRSC), MagNA Pure (99.7% using SM2), AL buffer (87.4% using S400HR), Cobas Omni LYS (97.0% using SM2), and NeuMoDx (93.4% using S400HR). For these reagents, filtered eluate was still cytotoxic when used undiluted in cell culture. However, CC_20_ values indicated that this remaining cytotoxicity would be removed by first or second (10^−1^ to 10^−2^) dilutions in the TCID_50_ assay, allowing evaluation of titer reduction using these reagents, with the caveat that the effective assay limit of detection (LOD) would be higher. Passing treated samples through more than one column or increasing the depth of the resin/bead bed within the spin column can also improve cytotoxicity removal for some reagents (unpublished data).

In addition to cytotoxicity removal, a successful filtration method must also purify virus without adversely affecting titer or integrity. We therefore assessed SARS-CoV-2 recovery after each filtration method. Using an input titer of 1.35 × 10^6^ TCID_50_/ml, triplicate purifications of virus through Sephadex LH-20 or Pierce detergent removal spin columns resulted in recovery of 100% of input virus (Fig. 1B). In contrast, the recoverable titer after one filtration through Amicon Ultra filters was 2.13 × 10^5^ TCID_50_/ml, an 84.5% reduction from input. Purification with S400HR and Bio-Beads SM2 matrices resulted in recoverable titers of 1.08 × 10^6^ TCID_50_/ml and 8.99 × 10^5^ TCID_50_/ml, losses of 20.1% and 33.6% of input virus, respectively.

### SARS-CoV-2 inactivation by specimen transport and molecular extraction reagents.

Specimen transport tubes are designed to inactivate microorganisms present in clinical specimens prior to sample transport while preserving the integrity of nucleic acids for molecular testing. If effective, these products have the potential to streamline SARS-CoV-2 diagnostic processing in testing laboratories by eliminating the requirement for CL3 processing or, for activities carried out at CL2, permitting processing outside an MSC. The BS EN 14476 standard requires demonstration of a >4-log_10_ titer reduction for virucidal suspension tests (24), and we were able to demonstrate a ≥4-log_10_ TCID_50_ titer reduction for all specimen transport media evaluated in a tissue culture fluid matrix (Table 2). However, infectious virus remained recoverable in treated samples after inactivation with most reagents tested (by either TCID_50_ assay or blind passage). The exceptions to this were PrimeStore MTM and 4 M GITC, from which no residual virus was detectable by either TCID_50_ assay or by the passaging of treated purified sample. While several contact times were evaluated for all of these reagents, length of contact time had no effect on either the level of virus titer reduction or whether virus remained detectable upon passage.

**TABLE 2:** Virus inactivation by specimen transport tube reagents

Reagent^*a*^	Reagent/virus ratio^*b*^	Contact time (min)	Titer reduction (log_10_ [±SE])	Virus detectable in titration^*c*^^,^^*d*^	Virus detectable in culture^*d*^
BioComma	10:1	10	4.9 (±0.2)	Yes (3/3)	Yes (3/3)
		30	4.9 (±0.2)	Yes (3/3)	Yes (3/3)
		60	4.8 (±0.2)	Yes (3/3)	Yes (3/3)
Sigma MM	1.5:1	10	≥4.8 (±0.1)	Yes (2/3)^†^	Yes (1/3)
		30	≥4.8 (±0.1)	Yes (1/3)^†^	Yes (1/3)
		60	≥4.8 (±0.1)	No (0/3)^†^	No (0/3)
eNAT	1:3	10	4.8 (±0.2)	Yes (3/3)	Yes (3/3)
		30	5.1 (±0.2)	Yes (3/3)	Yes (3/3)
		60	5.2 (±0.2)	Yes (3/3)	Yes (3/3)
	3:1	10	≥5.1 (±0.1)	No (0/3)*	Yes (1/3)
		30	≥5.1 (±0.1)	No (0/3)*	Yes (1/3)
		60	≥5.1 (±0.1)	No (0/3)*	No (0/3)
Primestore MTM	1:3	10	≥5.1 (±0.2)	No (0/3)*	No (0/3)
		30	≥5.1 (±0.2)	No (0/3)*	No (0/3)
		60	≥5.1 (±0.2)	No (0/3)*	No (0/3)
Cobas PCR medium	1:1.4	10	4.6 (±0.1)	Yes (3/3)	Yes (3/3)
		30	4.8 (±0.1)	Yes (3/3)	Yes (3/3)
		60	4.8 (±0.1)	Yes (3/3)	Yes (3/3)
Aptima specimen TM	5.8:1	10	≥4.4 (±0.1)	Yes (1/3)	No (0/3)
		30	≥4.4 (±0.1)	No (0/3)	No (0/3)
		60	≥4.4 (±0.1)	Yes (2/3)	Yes (1/3)
Virus transport and preservation medium (inactivated)	10:1	10	5.0 (±0.2)	Yes (3/3)	Yes (3/3)
		30	4.9 (±0.2)	Yes (3/3)	Yes (3/3)
		60	4.8 (±0.2)	Yes (3/3)	Yes (3/3)
DNA/RNA Shield	10:1	10	≥4.8 (±0.2)	No (0/3)**	No (0/3)
		30	≥4.8 (±0.2)	No (0/3)**	No (0/3)
		60	≥4.8 (±0.2)	No (0/3)**	No (0/3)
2 M GITC/Tx TM	10:1	30	≥4.6 (±0.1)	No (0/3)*	Yes (1/3)
4 M GITC/Tx TM	10:1	30	≥5.1 (±0.2)	No (0/3)*	No (0/3)
40% GHCl/Tx TM	10:1	30	≥4.6 (±0.1)	Yes (1/3)*	Yes (3/3)

a TM, transport medium; MM, molecular transport medium; GHCl, guanidine hydrochloride; GITC, guanidinium isothiocyanate; Tx, Triton X-100.

b All specimen transport media were evaluated in a tissue culture fluid matrix.

c Samples were titrated by TCID_50_, with a limit of detection of 5 TCID_50_/ml (0.7 log_10_ TCID_50_/ml) unless otherwise indicated as follows: *, limit of detection of 50 TCID_50_/ml (1.7 log_10_ TCID_50_/ml) due to cytotoxicity in neat wells of TCID_50_ assay; **, limit of detection of 504 TCID_50_/ml (2.7 log_10_ TCID_50_/ml) due to cytotoxicity in neat and −1 wells of TCID_50_ assay; †, titration by plaque assay with a limit of detection of 3.3 PFU/ml (0.5 log_10_ PFU/ml).

d Values in parentheses represent the number of positive samples/number of replicates.

We also sought to inform sample processing by examining inactivation by molecular extraction lysis buffers used in several manual and automated extraction protocols within SARS-CoV-2 diagnostic and research laboratories. We could demonstrate a ≥4-log_10_ reduction in TCID_50_ titer for all but two molecular extraction reagents when they were evaluated using tissue culture fluid (Table 3). The exceptions to this were AL and Cobas Omni LYS, where remaining cytotoxicity in the filtered eluate increased the TCID_50_ LOD to a level such that the maximum calculable titer reductions were ≥3.5 and ≥3.9 log_10_ TCID_50_, respectively. However, given that no virus was detected at any passage, it is likely that infectious virus was effectively inactivated by these two reagents. For reagents tested with multiple contact times (NucliSENS and Panther Fusion), shorter times (10 min) were as effective at reducing virus titers as longer contact times. Most reagents reduced viral titers to around the TCID_50_ assay LOD, indicating that any remaining virus posttreatment was present only at very low titers (<10 TCID_50_/ml), but higher levels of virus were recoverable from samples treated with some extraction buffers. For NeuMoDx lysis buffer, although titers were reduced by ≥4 log_10_ TCID_50_, an average of 91 (±38) TCID_50_/ml remained detectable. Similarly, buffer AVL reduced virus titers by 5.1 log_10_ TCID_50_, but after treatment virus was detectable in all treated sample replicates (average, 54 ± 18 TCID_50_/ml). However, after addition of four sample volumes of absolute ethanol following a 10-min contact time with AVL (the next step in the Qiagen Viral RNA Minikit manual), a ≥5.9-log_10_ titer reduction was recorded, with no virus recoverable following passages in cell culture.

**TABLE 3:** Virus inactivation by molecular extraction reagents

Reagent^*a*^	Virus matrix	Reagent/virus ratio	Contact time (min)	Titer reduction (log_10_ [±SE])	Virus detectable in titration^*c*^^,^^*d*^	Virus detectable in culture^*d*^
AVL	Tissue culture fluid	4:1	10	5.1 (±0.1)	Yes (3/3)	Yes (3/3)
AVL + ethanol	Tissue culture fluid	4:1:4^*b*^	10^*e*^	≥5.9 (±0.2)	No (0/3)	No (0/3)
RLT (+BME)	Ferret lung homogenate	9:1	10	≥4.9 (±0.2)	No (0/3)*	No (0/3)
MagNA Pure External LB	Tissue culture fluid	1:1	10	≥4.4 (±0.2)	No (0/3)*	No (0/3)
AL	Tissue culture fluid	1:1	10	≥3.5 (±0.2)	No (0/3)**	No (0/3)
Cobas Omni LYS	Tissue culture fluid	1:1	10	≥3.9 (±0.1)	No (0/3)**	No (0/3)
PHE in-house LB	Tissue culture fluid	4:1	10	≥5.6 (±0.1)	Yes (1/3)*	Yes (2/3)
E&O Lab VPSS	Tissue culture fluid	10:1	30	≥5.2 (±0.2)	No (0/3)*	Yes (2/3)
		1:1	10	≥5.1 (±0.1)	No (0/3)*	Yes (1/3)
E&O Lab LB	Tissue culture fluid	1:1	10	≥5.1 (±0.1)	No (0/3)*	No (0/3)
NeuMoDx LB	Tissue culture fluid	1:1	10	4.3 (±0.2)	Yes (3/3)*	Yes (3/3)
Samba II SCoV LB	Tissue culture fluid	1:1	10	4.8 (±0.1)	Yes (3/3)	Yes (3/3)
NucliSENS LB	Tissue culture fluid	1:1	10	≥5.0 (±0.1)	Yes (2/3)^†^	Yes (1/3)
			30	≥5.1 (±0.0)	No (0/3)^†^	Yes (1/3)
		2:1	10	≥4.9 (±0.1)	No (0/3)*	No (0/3)
Panther Fusion specimen lysis tubes	Tissue culture fluid	1.42:1	10	≥4.4 (±0.0)	No (0/3)^†^	No (0/3)
			30	≥4.4 (±0.0)	No (0/3)^†^	Yes (1/3)
			60	≥4.4 (±0.0)	No (0/3)^†^	Yes (1/3)
	Pooled swab material	1.42:1	30	≥5.1 (±0.1)	No (0/3)	No (0/3)

a LB, lysis buffer; BME, beta-mercaptoethanol; PHE, Public Health England.

b Values represent AVL/virus/ethanol.

c Samples were titrated by TCID_50_, with a limit of detection of 5 TCID_50_/ml (0.7 log_10_ TCID_50_/ml) unless otherwise indicated as follows: *, limit of detection of 50 TCID_50_/ml (1.7 log_10_ TCID_50_/ml) due to cytotoxicity in neat wells of TCID_50_ assay; **, limit of detection of 504 TCID_50_/ml (2.7 log_10_ TCID_50_/ml) due to cytotoxicity in neat and −1 wells of TCID_50_ assay; †, titration by plaque assay with a limit of detection of 3.3 PFU/ml (0.5 log_10_ PFU/ml).

d Values in parentheses represent the number of positive samples/number of replicates.

e Ethanol was added following a 10-min contact time with AVL.

Panther Fusion lysis buffer was further tested against a relevant clinical sample matrix, pooled fluid from oropharyngeal (OP) and nasopharyngeal (NP) swab specimens, resulting in a ≥5.1-log_10_ titer reduction with no remaining infectious virus detectable. We additionally evaluated the tissue lysis buffer RLT using homogenized ferret lung as sample material, with treatment resulting in a ≥4.8-log_10_ titer reduction with no residual infectious virus detectable.

### SARS-CoV-2 inactivation by detergents.

Detergents can be used to inactivate lipid enveloped viruses such as coronaviruses by disrupting the viral envelope, thereby rendering them unable to attach or enter cells (26–29). Here, we evaluated Triton X-100, SDS, NP-40, and Tween 20 for their ability to inactivate SARS-CoV-2. SDS treatment at 0.1% or 0.5% reduced titers by ≥5.7 and ≥6.5 log_10_ TCID_50_, respectively, while both concentrations of NP-40 reduced titers by ≥6.5 log_10_ TCID_50_ with no residual virus detectable following NP-40 treatment. In contrast, up to 0.5% Tween 20 had no effect on viral titers. Triton X-100 is commonly used in viral inactivation reagents, and here we show that at both 0.1% and 0.5% (vol/vol) concentrations, virus titers in tissue culture fluid were reduced by ≥4.9 log_10_ TCID_50_, even with less than 2 min of contact time (Table 4). Furthermore, we were unable to recover infectious virus from samples treated with 0.5% Triton X-100 for 10 min or longer. We also saw effective inactivation of SARS-CoV-2 by SDS, NP-40, and Triton X-100 in spiked NP and OP swab specimen fluid, but, importantly, we were not able to replicate this in spiked serum; 1% Triton X-100 reduced titers in human serum by only a maximum of 2 log_10_ TCID_50_ with contact times of up to 2 h.

**TABLE 4:** Virus inactivation by detergents

Detergent	Virus matrix	Detergent/virus ratio (%)^*a*^	Contact time (min)	Titer reduction (log_10_ [±SE])	Virus detectable in TCID_50_ assay^*b*^^,^^*c*^	Virus detectable in culture^*c*^	RNA integrity (*C_T_*)^*d*^
Tween 20	Tissue culture fluid	0.1	30	0.0 (±0.2)	Yes (3/3)	Yes (3/3)	ND
		0.5	30	0.0 (±0.2)	Yes (3/3)	Yes (3/3)	+0.2 (±0.0)
Triton X-100	Tissue culture fluid	0.1	30	≥4.9 (±0.1)	Yes (3/3)	Yes (3/3)	ND
		0.5	<2	5.9 (±0.2)	Yes (3/3)	Yes (3/3)	+0.1 (±0.2)
			10	≥6.2 (±0.2)	No (0/3)	No (0/3)	+1.4 (±0.1)
			30	≥6.1 (±0.2)	No (0/3)	No (0/3)	+3.6 (±0.1)
	Human serum	1.0	30	1.3 (±0.2)	Yes (3/3)	Yes (3/3)	ND
			60	1.5 (±0.2)	Yes (3/3)	Yes (3/3)	ND
			120	2.0 (±0.2)	Yes (3/3)	Yes (3/3)	ND
	Pooled swab material	0.5	30	≥6.1 (±0.2)	No (0/3)	Yes (1/3)	+8.3 (±0.2)
SDS	Tissue culture fluid	0.1	30	5.7 (±0.1)	Yes (3/3)	Yes (3/3)	+1.3 (±0.2)
		0.5	30	≥6.5 (±0.1)	Yes (1/3)	Yes (2/3)	−0.6 (±0.2)
	Pooled swab material	1.0	30	5.7 (±0.2)	Yes (3/3)	Yes (2/3)	+6.1 (±0.0)
NP-40	Tissue culture fluid	0.1	30	≥6.5 (±0.1)	No (0/3)	No (0/3)	+9.0 (±0.2)
		0.5	30	≥6.5 (±0.1)	No (0/3)	No (0/3)	+10.3 (±0.1)
	Pooled swab material	0.5	30	≥6.1 (±0.2)	No (0/3)	No (0/3)	+8.7 (±0.1)

a Values represent volume/volume.

b The limit of detection in TCID_50_ assay was 5 TCID_50_/ml (0.7 log_10_ TCID_50_/ml).

c The values in parentheses represent the number of positive samples/number of replicates.

d The difference in the *C_T_* value in SARS-CoV-specific real-time RT-PCR and that in the PBS-treated control (± standard error). ND, not done.

In addition to evaluating inactivation efficacy by detergents, we assessed the effects of treatment on RNA integrity to determine their suitability for inactivation prior to nucleic acid testing. Extracted RNA from treated samples was tested using a SARS-CoV-2-specific qRT-PCR, and the *C_T_* difference between detergent-treated samples and mock-treated controls was determined (Table 4). A time-dependent increase in *C_T_* value following treatment with 0.5% Triton X-100 was observed, indicating a detrimental effect on RNA stability with increasing treatment times. Treatment with NP-40 had a marked effect, with a 30-min treatment leading to an increase in 9 to 10 *C_T_*s. While we saw no increase in *C_T_* in tissue culture fluid samples treated with 0.5% SDS, we observed an increase in the *C_T_* value for SDS-treated swab fluid samples, likely due to an increased concentration of RNases in clinical samples.

### SARS-CoV-2 inactivation by other chemical treatments.

Fixation and inactivation of viruses by the addition of formaldehyde or of a combination of formaldehyde and glutaraldehyde is a well-established protocol, particularly for diagnostic electron microscopy (30, 31). A treatment with 4% or 2% formaldehyde for 15 or 60 min reduced virus titers by ≥4.8 log_10_ TCID_50_ when evaluated against a tissue culture fluid matrix, with no remaining infectious virus detectable (Table 5). When infected monolayers were subjected to the same treatment protocol, all titer reductions were ≥6.8 log_10_ TCID_50_, with 60 min of contact time moderately more effective than 15 min. However, in this format, a 60-min 4% formaldehyde treatment was the only one from which no infectious virus was detectable. A mixture of 2% formaldehyde and 1.5% glutaraldehyde tested on infected monolayers reduced virus titers by ≥6.7 log_10_ TCID_50_, with no remaining infectious virus detectable for both 15- and 60-min contact times. Polyhexanide biguanide (PHMB) is a polymer used as a disinfectant and antiseptic, evaluated here as a potential lysis buffer, but it was able to reduce viral titers by only 1.6 log_10_ TCID_50_ at the highest concentration tested (2%).

**TABLE 5:** Other reagent types

Reagent	Virus matrix	Reagent/virus ratio or % (vol/vol)	Contact time (min)	Titer reduction (log_10_ [±SE])	Virus detectable in TCID_50_^*b*^^,^^*c*^	Virus detectable in culture^*c*^
Formaldehyde	Tissue culture fluid	4%	15	≥4.8 (±0.2)	No (0/3)	No (0/3)
			60	≥5.0 (±0.2)	No (0/3)	No (0/3)
		2%	15	≥4.8 (±0.2)	No (0/3)	No (0/3)
			60	≥5.0 (±0.2)	No (0/3)	No (0/3)
	Infected monolayer	4%	15	≥6.9 (±0.2)	Yes (1/3)	Yes (1/3)
			60	≥7.5 (±0.2)	No (0/3)	No (0/3)
		2%	15	≥6.8 (±0.2)	Yes (2/3)	Yes (2/3)
			60	≥7.3 (±0.2)	Yes (2/3)	Yes (3/3)
Formaldehyde + glutaraldehyde	Tissue culture fluid	2% + 1.5%	60	≥5.0 (±0.2)	No (0/3)	No (0/3)
	Infected monolayer	2% + 1.5%	15	≥6.7 (±0.1)	No (0/3)	No (0/3)
			60	≥6.7 (±0.1)	No (0/3)	No (0/3)
Methanol^*a*^	Infected monolayer	100%	15	≥6.7 (±0.1)	No (0/3)	No (0/3)
PHMB						
0.1%	Tissue culture fluid	10:1	30	1.4 (±0.2)	Yes (3/3)	Yes (3/3)
1.0%	Tissue culture fluid	10:1	30	1.5 (±0.2)	Yes (3/3)	Yes (3/3)
2.0%	Tissue culture fluid	10:1	30	1.6 (±0.2)	Yes (3/3)	Yes (3/3)

a Ice-cold methanol.

b The limit of detection in TCID_50_ assay was 5 TCID_50_/ml (0.7 log_10_ TCID_50_/ml).

c The values in parentheses represent the number of positive samples/number of replicates.

## DISCUSSION

Samples containing infectious SARS-CoV-2 require an initial inactivation step in primary containment (e.g., in an MSC) before further processing; given the rapid emergence of SARS-CoV-2, these inactivation protocols have been guided by existing data for other coronaviruses, and there is an urgent need to both confirm these historical data using the new virus and to validate new approaches for inactivating SARS-CoV-2. We therefore analyzed numerous commercially and commonly available reagents used by public health agencies and research laboratories around the world in their response to the pandemic. In addition, to address challenges of reagent cytotoxicity in inactivation evaluation, we provide data on the effectiveness of filtration methods for removing cytotoxicity from chemically treated samples.

Knowledge of the expected amount of infectious virus in clinical samples obtained from COVID-19 patients is important when viral inactivation study data are applied to diagnostic sample processing, allowing end users to interpret whether the material they are handling is likely to represent an infectious risk to themselves and others. These values are dependent on several factors, including time post-symptom onset, duration of symptoms, time elapsed between sampling and testing, the presence of neutralizing antibody responses, and immunocompetency of the individual (32). Data regarding quantitative infectious viral levels in typical clinical specimens are minimal, with most studies reporting viral loads as determined by qRT-PCR only (33–35). However, one study investigating infectious titers in 90 qRT-PCR-positive NP or endotracheal samples from COVID-19 patients estimated a median titer of 3.3 log_10_ TCID_50_/ml (32). Although here we were able to demonstrate a >4-log_10_ reduction in titer for all specimen transport reagents, the observation that virus could be recovered from most treated samples indicates that while these reagents can effectively reduce viral titers, they cannot be assumed to completely inactivate SARS-CoV-2 in clinical specimens.

Limited SARS-CoV-2 inactivation data on molecular extraction reagents used in nucleic acid detection assays are currently available. We demonstrate here that the majority of commonly used reagents evaluated were effective at reducing viral titers by more than 4 logs, with several treatments completely removing all infectivity. For two reagents, buffer AL and Cobas Omni LYS buffer, we were not able to show a >4-log reduction. However, this was due to an increase in the effective limit of detection in the TCID_50_ assay as no purification system was able to remove all of the cytotoxicity. Given that no virus was detected in serial passage of the treated samples, it is probable that treatment with either of these buffers is effective at inactivating SARS-CoV-2. A previous study reported that buffer AVL either alone or in combination with ethanol was not effective at completely inactivating SARS-CoV-2 (17). In contrast, we could not recover any infectious virus from samples treated with AVL plus ethanol, consistent with previous studies indicating that AVL and ethanol in combination is effective at inactivating MERS and other enveloped viruses (10, 36) and indicating that both AVL and ethanol steps of manual extraction procedures should be performed before removal of samples from primary containment for additional assurance. Our detergent inactivation data indicating that SDS, Triton X-100, and NP-40, but not Tween 20, can effectively inactivate SARS-CoV-2 both in tissue culture fluid and also in pooled NP and OP swab fluid, which more accurately mimic authentic clinical specimen types, corroborate findings of a recent study (19). However, as has been demonstrated for other viruses (33), we observed an inhibitory effect of serum on virus inactivation by detergent, highlighting the importance of validating inactivation methods with different sample types.

Based on our findings comparing filtration matrices, we found that the optimum method for reagent removal for inactivation studies is determined by evaluating three factors: (i) effectiveness of cytotoxicity removal, (ii) efficiency of virus recovery, and (iii) the ease of performing these methods within a containment space. Methods permitting complete removal of cytotoxic reagent components with no or little effect on virus recovery give assurance that low levels of residual virus, if present, could be detected in virus inactivation studies. During reagent testing, there were several instances where we noted residual cytotoxicity in the neat eluate, which is contrary to what was expected based on the initial reagent removal data and is likely due to the extended incubation period required for inactivation testing (up to 7 days, compared with overnight for cytotoxicity evaluation). In all cases, however, we were still able to enhance the levels of titer reduction detectable compared with what would have been achieved by sample dilution alone.

In conclusion, we have evaluated methods for straightforward, rapid determination of purification options for reagents prior to inactivation testing, enabling establishment of effective methods for sample purification while minimizing virus loss. This is applicable to inactivation studies for all viruses (known and novel), not only SARS-CoV-2. We have applied these methods to obtain SARS-CoV-2 inactivation data for a wide range of reagents in use (or proposed for use) in SARS-CoV-2 diagnostic and research laboratories. In addition to guiding laboratory risk assessments, this information enables laboratories to assess alternative reagents that may be used for virus inactivation and nucleic acid extraction, particularly considering concerns about extraction reagent availability due to increased global demand caused by the COVID-19 pandemic. Furthermore, chemical treatments evaluated here are commonly used for inactivation of a wide range of different viruses and other pathogens, and the results presented may be used to directly inform and improve the design of future inactivation studies.

## Supplementary Material

Supplemental file 1
