# Supplementary material for: Analysis of Inactivation of SARS-CoV-2 by Specimen Transport Media, Nucleic Acid Extraction Reagents, Detergents, and Fixatives
Source: J Clin Microbiol. 2020 Oct 21;58(11):e01713-20. doi: 10.1128/JCM.01713-20 (PMC7587104; doi:10.1128/JCM.01713-20)
Supplement: Supplemental file 1 [file JCM.01713-20-s0001.pdf]

Supplementary Table 1: Reagent Details

Reagent Type	Reagent	Manufacturer Cat#	Reagent composition	Recommended ratio of sample to reagent	Recommended contact time
	Virus Transport and Preservation Medium (Inactivated)	BioComma Ltd. #YMJ-E	Not known	Swab placed directly into tube containing 3mL reagent	None given
	Sigma MM	Medical Wire #MWMM	Guanidine thiocyanate, Ethanol (concentrations unknown)	Up to 1 vol sample to 1.5 vols reagent (up to 0.67:1)	None given
	eNAT	Copan #608CS01R	42.5-45% guanidine thiocyanate, detergent, Tris-EDTA, HEPES.	Swab placed directly into tube containing 1 or 2mL reagent. For urine, 3:1	None given
	Primestore	Longhorn #PS-MTM-3	<50% guanidine thiocyanate, <23% ethanol	1:3	None given
Specimen Transport Tube Reagents	Cobas PCR	Roche #08042969001	≤40% guanidine hydrochloride, Tris-HCl	Swab placed directly into tube	None given
	Aptima Specimen Transport Medium	Hologic #PRD-03546	Not known	Swab OR 0.5mL VTM sample added to tube containing 2.9mL buffer	None given
	DNA/RNA Shield	Zymo Research #R1100	Not known	1:3	None given
	40% GHCL/Tx TM	Oxoid/Thermo Fisher #EB1351A	28.3% guanidine hydrochloride, 2.1% Triton X-100, Tris-EDTA	Swab placed directly into tube	None given
	2M GITC/Tx TM	Oxoid/Thermo Fisher #EB1349A	18.9% guanidine thiocyanate, 2.4% Triton X-100, Tris-EDTA	Swab placed directly into tube	None given
	4M GITC/Tx TM	Oxoid/Thermo Fisher #EB1350A	31.8% guanidine thiocyanate, 2.0% Triton X-100, Tris-EDTA	Swab placed directly into tube	None given
	NucliSENS Lysis Buffer	Biomerieux #200292	50% guanidine thiocyanate, <2% Triton X-100, <1% EDTA	1:2-1:200	10 mins
	Panther Fusion	Hologic #PRD-04339	Not known	1:1.42	
	Buffer AVL	QIAGEN #19073	50-70% guanidine thiocyanate	1:4	10 mins
	MagNA Pure 96 External Lysis Buffer	Roche #06374913001	30-50% guanidine thiocyanate, 20-25% Triton X-100, <100mM Tris-HCl, 0.01% bromophenol blue.	1:1	None given
	Buffer AL	QIAGEN #19075	30-50% guanidine hydrochloride, 0.1-1% maleic acid	1:1	None given
Molecular Extraction Reagents	Cobas Omni LYS	Roche #06997538190	30-50% guanidine thiocyanate, 3-5% dodecyl alcohol, ethoxylated, 1-2.5% dithiothreitol	No instructions for use as off-board lysis buffer	None available
	PHE in-house LB	PHE Media Services	96.6% guanidine thiocyanate, 1.9% Triton X-100, Tris-EDTA	None available	None available
	Buffer RLT	QIAGEN #79216	30-50% guanidine thiocyanate	Tissue to be homogenized directly in undiluted buffer	None given
	NeuMoDx Viral Lysis Buffer	NeuMoDx Molecular,Inc. #401600	<50% guanidine hydrochloride, <5% Tween 20, <1% EDTA, <0.1% sodium azide	1:1	None given
	VPSS	E&O Laboratories #BM1675	Not known	Not known	Not known
	Lysis Buffer	E&O Laboratories #BM1676	Not known	Not known	Not known

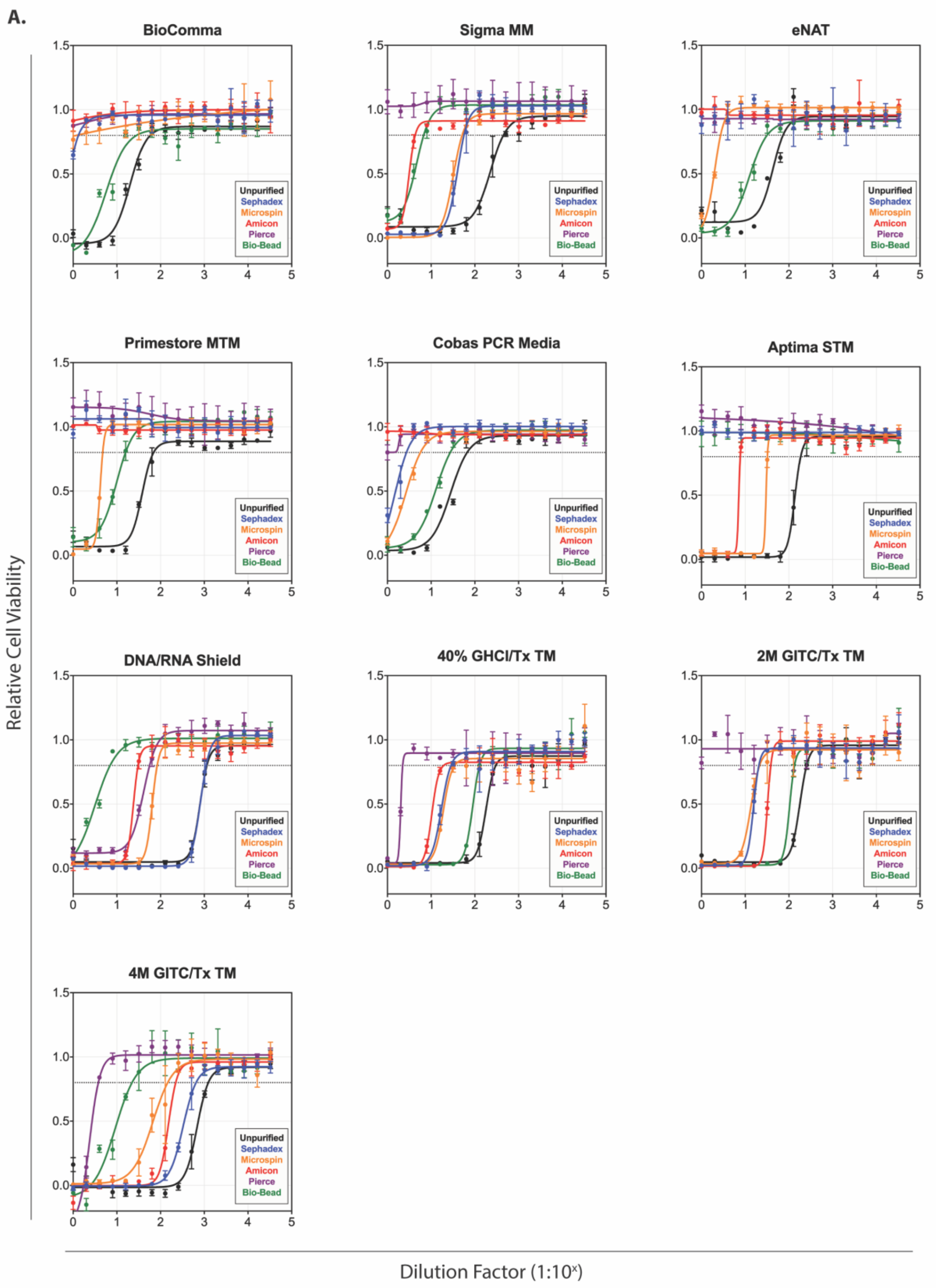
A.
BioComma
Sigma MM
eNAT
Primestore MTM
Cobas PCR Media
Aptima STM
DNA/RNA Shield
40% GHCl/Tx TM
2M GITC/Tx TM
4M GITC/Tx TM
Unpurified
Sephadex
Microspin
Amicon
Pierce
Bio-Bead
Relative Cell Viability
Dilution Factor (1:10ˣ)

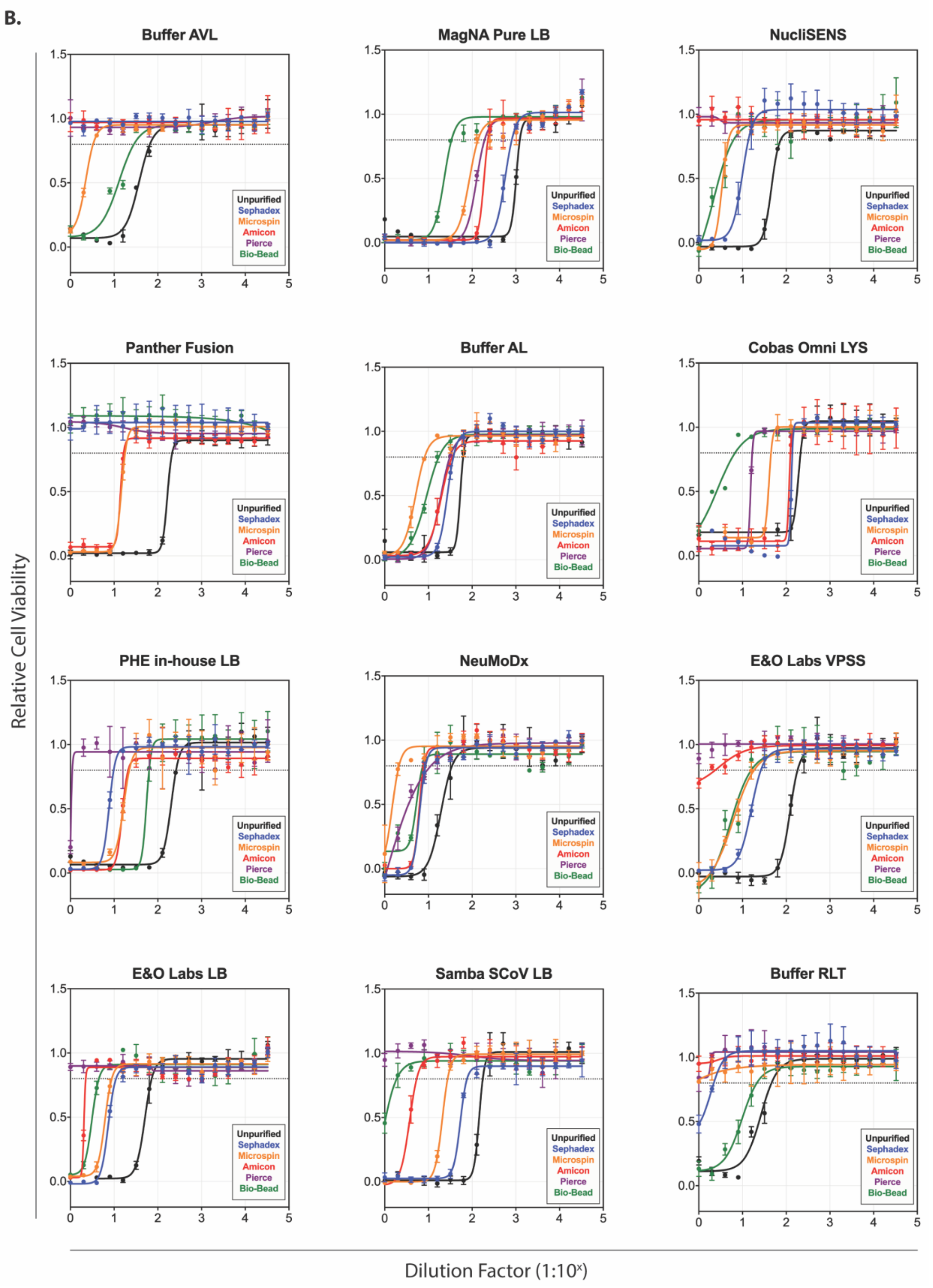
B.
Buffer AVL
MagNA Pure LB
NucliSENS
Panther Fusion
Buffer AL
Cobas Omni LYS
PHE in-house LB
NeuMoDx
E&O Labs VPSS
E&O Labs LB
Samba SCoV LB
Buffer RLT
Unpurified
Sephadex
Microspin
Amicon
Pierce
Bio-Bead
Relative Cell Viability
Dilution Factor (1:10ˣ)

C.

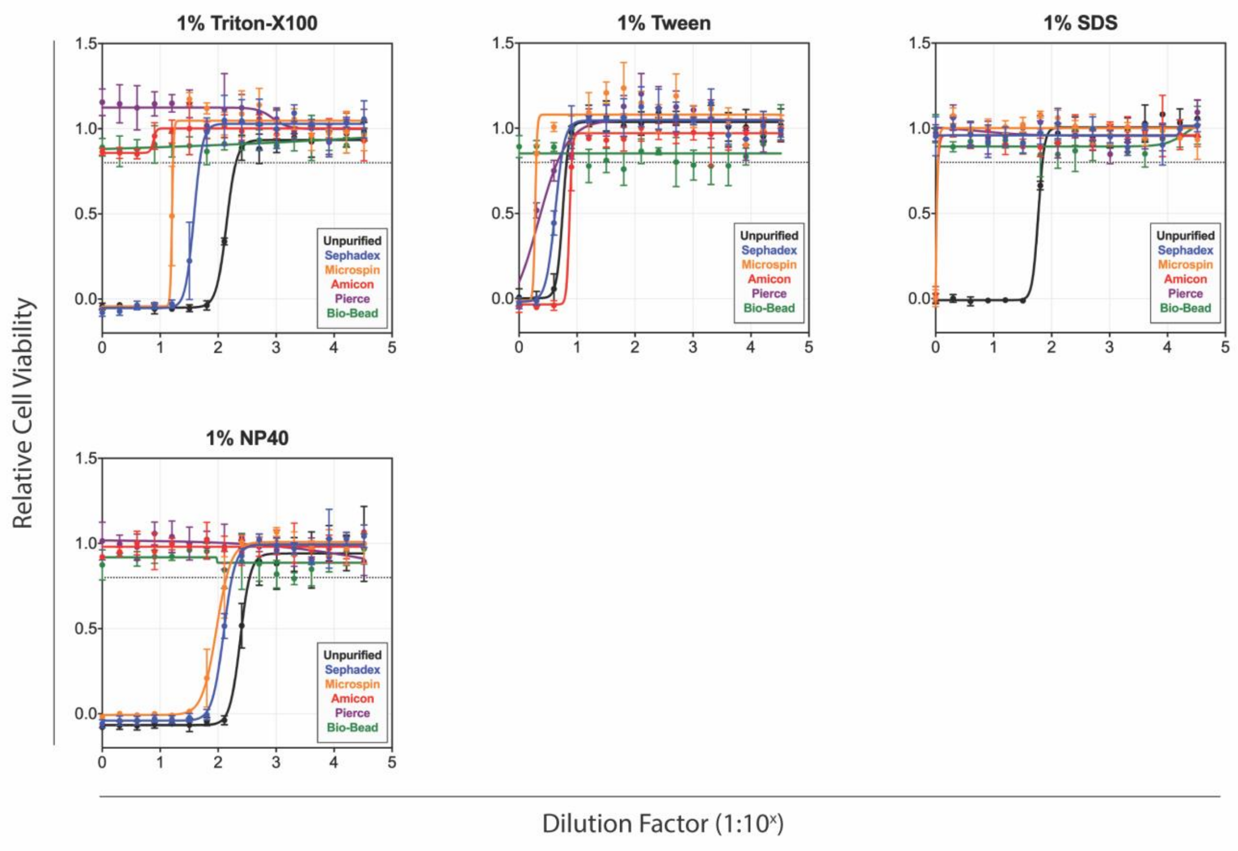
1% Triton-X100
1% Tween
1% SDS
1% NP40
Unpurified
Sephadex
Microspin
Amicon
Pierce
Bio-Bead
Relative Cell Viability
Dilution Factor (1:10^x)

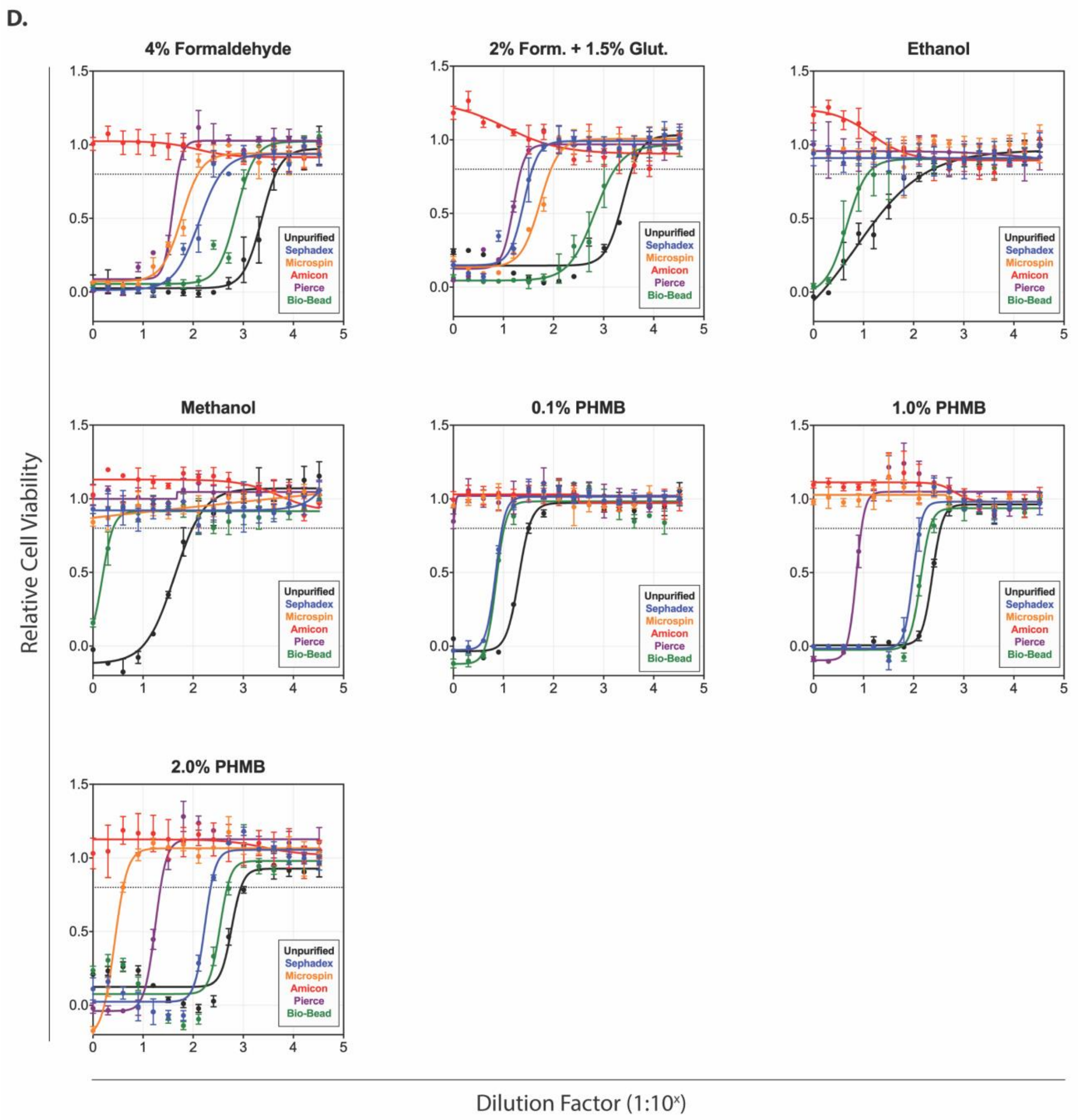

Supplementary Figure 1: Cytotoxicity of virus inactivation reagents after passing through purification matrices. Concentration-response curves in Vero cells treated with a 2-fold serial dilution of reagent. At 24 h post treatment cell viability was determined, with values normalized to mock treated cells. Each point represents the mean of triplicate wells, with error bars indicating standard deviation. Graphs are representative of at least 2 independent experiments. Matrices used: Sephadex LH-20 (blue); Sephacryl S400HR (orange); Amicon Ultra 50kDa molecular weight cut off (red); Pierce detergent removal spin columns (DRSC) (purple); or Bio-Bead SM2 (green). **(A)** Reagents used in specimen transport tubes: GHCl - guanidine hydrochloride; GITC - guanidinium isothiocyanate; Tx – Triton X-100; TM – Transport Medium **(B)** Reagents used in molecular extraction protocols: PHMB - polyhexamethylene biguanide. **(C)**

Detergents commonly used for virus inactivation: SDS - sodium dodecyl sulfate; NP40 - nonyl phenoxypolyethoxylethanol. **(D)** Other reagents commonly used for virus inactivation.
